# Easy Synthesis of a Multifunctional Macrophotoinitiator with Pendant Moieties of Benzoin Methyl Ether Derivative for Use as Active Surface-Modifier of Inorganic Fillers

**DOI:** 10.3390/polym18101265

**Published:** 2026-05-21

**Authors:** Halyna Ohar, Maria Tokareva, Viktor Tokarev

**Affiliations:** 1 Department of Occupational and Life Safety, Lviv Polytechnic National University, 12 S. Bandery, 79013 Lviv, Ukraine; mariia.o.tokareva@lpnu.ua; 2 Department of Organic Chemistry, Lviv Polytechnic National University, 12 S. Bandery, 79013 Lviv, Ukraine; viktor.s.tokariev@lpnu.ua

**Keywords:** multifunctional photoinitiator, copolymer of maleic anhydride, benzoin derivative-based macrophotoinitiator, polymer modification reaction, macrophotoinitiator decomposition, surface modification of inorganic fillers, polymer adsorption, graft polymerization

## Abstract

A novel macromolecular photoinitiator (MPI) was synthesized from a copolymer of maleic anhydride and methyl methacrylate and subsequently functionalized with 3-hydroxy-2-methoxy-1,2-diphenylpropan-1-one moieties via a polymer-analogous acylation reaction. The structure and physicochemical properties of the MPI were characterized by IR, UV–Vis, NMR, DSC, and TGA analyses. TiO_2_ nanoparticles were successfully functionalized with the MPI, yielding materials with enhanced surface activity and photoinitiating efficiency. The MPI-modified TiO_2_ facilitated efficient UV-induced polymerization of methyl methacrylate, as confirmed by DLS and SEM analyses. Compared with unmodified fillers, the resulting composites exhibited improved dispersion, accelerated polymerization rates, and enhanced mechanical properties. This hybrid strategy offers a promising approach for the development of high-performance polymer nanocomposites through the integration of surface-engineered inorganic fillers and photoreactive polymers.

## 1. Introduction

Over the past decades, photoinitiated reactions have attracted considerable attention in polymer chemistry due to their widespread application in various industrial fields, including the manufacture of coatings, adhesives, microelectronic devices, 3D printing, optics, and dental materials [1,2,3,4,5]. The nature of the UV initiator and the type of UV initiation system are crucial, as they determine the curing rate and significantly influence the overall performance of the cured materials, including yellowing, odor, and migration [1,3,6,7,8,9]. Low-molecular-weight photoinitiators, such as benzoin, acetophenone, α-hydroxyketones, benzophenone, thioxanthone, and their derivatives, have been widely used others [4,7,10,11,12,13]. However, their inherent disadvantages, including relatively strong odor and high migration from cured materials, significantly limit their applications [7,14]. In particular, unreacted low-molecular-weight photoinitiator (PI) molecules and their photolysis products can migrate to the surface of post-cured materials or be extracted into liquid-packaged goods when the UV-cured materials are used in food packaging applications [15,16,17,18]. Tethering PI molecules to polymer backbones, thereby forming macromolecular photoinitiators, represents a promising strategy to overcome these limitations.

Macrophotoinitiators (MPIs) are defined as reactive polymers containing pendant, in-chain, or terminal photoactive chromophores [19,20]. Upon light absorption, these chromophores generate active species capable of initiating the polymerization and crosslinking of mono- and multifunctional monomers and oligomers. MPIs bearing pendant photoinitiating moieties are generally more efficient—and therefore preferable—for polymer curing compared to those containing in-chain groups. The latter are of greater interest for the synthesis of block-copolymers [21,22]. MPIs with terminal photoinitiating moieties typically exhibit a relatively low chromophore content.

The main advantages inherent to MPIs, owing to their high molecular weight, include a low tendency to migrate in both uncured formulations and UV-cured materials; low volatility, which helps reduce formulation odor; reduced yellowing due to the limited migration of residual unreacted PI and its photodecomposition products to the material surface; and good compatibility with other components of photopolymer formulations [23,24,25,26].

MPIs are attracting increasing interest as promising initiators of radical reactions due to the high reaction rates they enable, their applicability at room temperature, and the simplicity of localized initiation in selected areas. These features open new opportunities for the development of advanced composite and photolithographic materials. Moreover, unlike their low-molecular-weight analogues, MPIs can exhibit not only initiating activity, but also surface-active and adsorption properties, and may serve as thickeners, stabilizers, and surface modifiers. Therefore, they represent promising candidates for the development of novel polymer composites for applications in medicine, the electronic industry, membrane technologies, and related fields.

The most widespread MPIs are those which generate active moieties via a free-radical mechanism. Photoinitiators (PIs) are typically classified into two types to the pathway of free-radical generation [1,4]. Type I photoinitiators, also known as α-cleavage or one-component photoinitiators, produce radicals through unimolecular bond cleavage upon irradiation. In contrast, Type II photoinitiators, also referred to as hydrogen-abstraction or two-component systems, generate radicals through bimolecular interactions with a co-initiator.

Type I MPIs are represented by polymers incorporating structural fragments of benzoin ethers [27,28,29,30,31], trichloroacetophenone and α-aminoketones [32,33]. Benzoin ether derivatives are well known and widely used Type I photofragmenting PIs. Upon light irradiation, they undergo α-cleavage to generate benzoyl and α-alkoxybenzyl primary radicals [4,34]. Among these, the benzoyl radicals act as the primary initiating species, whereas the α-alkoxybenzyl radicals predominantly behave as chain-terminating agents. In the presence of substrate containing labile hydrogen atoms, benzoyl radicals can abstract hydrogen to generate new initiating radical on the substrate molecules, facilitating grafting or crosslinking reactions. Upon irradiation, MPIs based on benzoin ether derivatives produce both polymer-bound and low-molecular-weight primary radicals. Depending on the method used to tether the benzoin ether fragment to the polymer backbone, the polymer-bound radical may be either benzoyl or α-alkoxybenzyl, while the low-molecular-weight radical is formed in a complementary manner [31,35].

MPIs of Type II are based on aromatic ketones and quinone, mostly benzophenone, thioxanthone, anthraquinone, and camphorquinone [11,14,36,37]. These MPIs generally function in the presence of a hydrogen-donating co-initiator, most commonly an amine, forming a charge-transfer complex. This is followed by proton transfer, generating an initiating aminoalkyl radical and a ketyl-type radical on the ketone. The ketyl radical primarily acts as a chain-terminating species. In some cases, hydrogen donors other than amines can also be employed [34].

There are two main approaches for the synthesis of MPIs bearing pendant photoinitiator (PI) groups. The first approach [37,38,39] involves a two-step process: (i) the preparation of polymerizable derivatives of low-molecular-weight photoinitiators by introducing unsaturated, preferably (meth)acrylic, substituents to form monomer-photoinitiators; and (ii) polymerization or copolymerization of these monomer-photoinitiators with other monomers to produce the final MPI.

The second approach involves polymer modification reactions to MPI synthesis with pendant photoactive moieties. In this method, reactive groups on molecules of low-molecular-weight PIs react with complementary functional groups on polymer backbones. For example, hydroxyl groups in photoinitiator molecules can react with anhydride functionalities in maleic anhydride or with acyl chloride groups in methacryloyl-containing copolymers. This technique was first applied by Kurusu [27] and subsequently developed by Angiolini et al. [35,40,41,42], as well as in our previous studies [43,44,45]. The approach is straightforward, affords high yields, and allows tuning of the MPI properties through the choice of low-molecular-weight PI and polymer carrier.

Strongly motivated by the development of the MPIs, we have developed a simple method for obtaining polymer photoinitiators with attached benzoin ether moieties using a polymer modification reaction. The feasibility of tethering benzoin fragments to macromolecules of the poly(methyl methacrylate-co-maleic anhydride) copolymer (p(MMA-co-MA)) via a polymer modification reaction based on the acylation mechanism of alcohol by anhydride group of p(MMA-co-MA) was demonstrated in our previous works [43,44,45].

The aim of this work was to synthesize new MPIs based on benzoin methyl ether and copolymers of maleic anhydride with methacrylates, as well as to investigate their properties and evaluate their potential for application in the surface modification of mineral nanofillers via “grafting from” polymerization of vinyl monomers. This approach will enable the design of the structure of a polymer covering (shell) on the surface of nanoparticles, imparting the desired properties, such as wettability, dispersibility, colloidal stability, affinity toward diverse polymer matrices, etc.

The starting materials for the MPI synthesis were selected considering the following factors: (i) benzoin methyl ether is one of the most applied photoinitiators due to its high efficiency, low cost, and availability; (ii) copolymers of maleic anhydride are also inexpensive and readily available and, more importantly, they are highly reactive, readily participate in polymer modification reactions, and exhibit high affinity toward various mineral surfaces.

## 2. Materials and Methods

Maleic anhydride (MA), methyl methacrylate (MMA), benzoin methyl ether (BME), 4 dodecylbenzenesulfonic acid (DBSA) and dimethyl sulfoxide (DMSO) were supplied by Sigma-Aldrich Co., Merck KGaA, Darmstadt, Germany.

Maleic anhydride (Aldrich) was recrystallized from chloroform. MMA was washed with an aqueous solution of sodium hydroxide to remove the inhibitor, dried over MgSO_4_, and distilled under reduced pressure. Ethyl acetate (EtAc), hexane, o-xylene, ethanol (EtOH), and methanol (MeOH), as well as other organic solvents—all of reagent grade, obtained from diverse suppliers (used as purchased)—were purified before their use, as reported by Weissberger et al. [46].

Titanium dioxide (TiO_2_) supplied by Sigma-Aldrich (product No. 718467) had a specific surface area of 37 m^2^/g (measured by nitrogen adsorption method) and an average particle size of 21 nm. Product 718467 exhibited a mixed rutile/anatase phase composition of 80%/20%.

### 2.1. Syntheses

#### 2.1.1. Synthesis of the 3-Hydroxy-2-methoxy-1,2-diphenylpropan-1-one (PI)

**Scheme 1:**
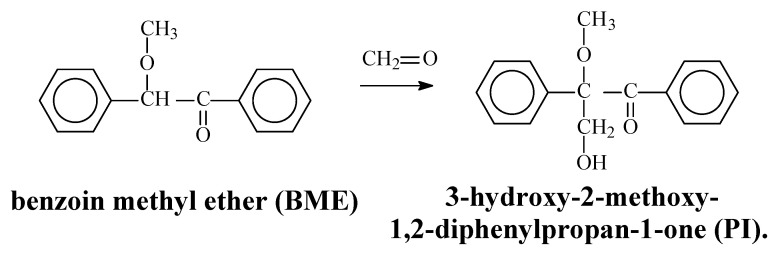
Synthesis of 3-hydroxy-2-methoxy-1,2-diphenylpropan-1-one from benzoin methyl ether.

PI was prepared according to the modified procedure of Hoffmann et al. (Scheme 1) [47]. In our case, the process was performed as follows: The suspension of paraformaldehyde in DMSO (1:7.5 by weight) was added to a stirred solution of BME in DMSO (1:1 by weight) at 30 °C. BME and paraformaldehyde were used in a weight ratio of 5:1; specifically, 10 g of BME and 2 g of paraformaldehyde were introduced into the reaction mixture. Then, 0.12 g of KOH dissolved in 2.0 g of EtOH was added to adjust the pH value to 8–9, and the temperature was raised to about 50 °C. The batch became neutral after about an hour. The reactive mixture was stirred for another 20 h at RT, and the product was precipitated by pouring into 60 mL of water. The precipitate was rinsed with 20 mL of water; after complete sedimentation at the bottom, it was subsequently decanted and extracted by the addition of 20 mL of benzene. The organic phase was separated, washed with water once more, dried over anhydrous sodium sulfate, and filtered out; afterward, benzene was distilled out under vacuum to obtain the final product. The PI was dried under vacuum at room temperature in the dark until constant mass was achieved. After drying, the samples were stored in a desiccator before analysis. The yield of PI was 12.3 g (72%).

#### 2.1.2. Synthesis of the Poly(methyl methacrylate-co-maleic anhydride) (p(MMA-co-MA))

Equimolar ratio of MMA and MA in EtAc was copolymerized using 2 wt.% lauryl peroxide as an initiator and 10 wt.% dodecyl mercaptan as a chain transfer agent at 60 ± 1 °C. The process was carried out in three-neck flask fitted with reflux condenser, mechanical stirrer, and inert-gas inlet. Copolymerization time was 8 h and the total conversion of the monomers reached 83%. The p(MMA-co-MA) copolymer was purified by twice precipitation in hexane and dried till constant weight at RT under reduced pressure. The molar fraction of MMA and MA units in the purified p(MMA-co-MA) copolymer (calculated from results of elemental analysis and the potentiometric back titration of –COOH groups) was 0.507 and 0.493, respectively. The intrinsic viscosity of p(MMA-co-MA) determined in acetone solution at 25 °C was [η] = 0.02 dL/g; and its *M*_n_ = 11,500 g/mol, *M*_w_ = 24,900 g/mol, *Đ* = 2.16 as determined by gel permeation chromatography.

#### 2.1.3. Synthesis of MPI via Tethering 3-Hydroxy-2-methoxy-1,2-diphenylpropan-1-one (PI) to p(MMA-co-MA)

**Scheme 2:**
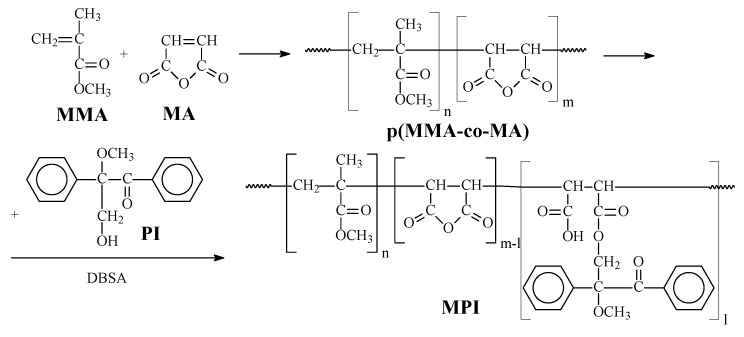
Synthesis of MPI.

Tethering of PI molecules to the polymer carrier p(MMA-co-MA) was achieved via a polymer modification reaction (Scheme 2), involving acylation of PI hydroxyl groups by MA units of the copolymer at a PI:MA molar ratio of 1:10. The reaction was conducted in the presence of DBSA as a catalyst (0.37 mol/L; 1.41 mL, 5.22 × 10^−4^ mol), maintaining a constant polymer-to-catalyst ratio. The selected conditions ensured efficient acylation while minimizing side reactions. The process was carried out in a sealed glass tube in 5 mL o-xylene as a solvent at temperature of 130 ± 1 °C for different times. MPI samples synthesized for different times were referred to as the MPI-N, where number N corresponds to the synthesis time. The products were dissolved in ethyl acetate and precipitated in water. The obtained raw MPIs were purified via twice precipitation into methanol and dried at 40 °C till constant weight at RT under reduced pressure in darkness. After drying, the samples were stored in a desiccator before analysis.

### 2.2. Analysis

#### 2.2.1. FTIR-Spectroscopy

FTIR spectra of thin polymer films of the initial p(MMA-co-MA) copolymer and MPI, applied on KBr plates, were recorded on a Specord M-80 spectrophotometer (VEB Carl Zeiss, Jena, Germany) within a range of 3700–600 cm^−1^.

#### 2.2.2. NMR Spectroscopy

Proton magnetic resonance ^1^H-NMR spectra of p(MMA-co-MA) and MPI were recorded on a 400 MHz VNMRS Varian NMR spectrometer with DMSO-d_6_ as the solvent. DMSO-d_6_ was used as an internal standard. Chemical shift of protons signals with a multiple character was determined by evaluation of positions of centers of symmetry of the signals.

#### 2.2.3. UV–Vis Spectroscopy

UV–Vis spectra of BME, PI, p(MMA-co-MA) copolymer and MPI in EtOH solutions placed into quartz cuvettes with 1 mm path length were recorded with a Shimadzu UV–1650 PC within a range of 200–350 nm.

The absorbance spectroscopic techniques are based on the Beer–Lambert Law:

*A* = *ε*·*b*·*C* (1)

where *A* is the absorbance of a photoinitiator solution; *ε* is the molar absorptivity (extinction coefficient) of a solute; *b* is the optical path length, in our case *b* = 1 mm; and *C* is the molar concentration of the solute (absorbing species), in mol/L.

The concentration of chromophoric groups (i.e., bonded PI moieties) of MPI in a solution (*C_PI_*, in mol/L) was determined using Equation (2):

(2) $$C_{PI}=\frac{A_{max}}{b\cdot \varepsilon }$$

where *A_max_* is the value of the max absorbance of a PI solution at λ = 252 nm.

Then, the content of the bonded PI moieties in the MPI structure (*W*) was calculated using Equation (3):

(3) $$W=\frac{C_{X}\cdot M_{PI}}{C_{MPI}}\cdot 100\%$$

where *W* is the content of the bonded PI moieties, in wt.%; *C_MPI_* is the concentration of MPI in EtOH solution, in mg/mL; *M_PI_* is the molecular weight of PI moieties, *M_PI_* = 216 g/mol; and *C_X_* is the concentration of low-molecular-weight photoinitiator moiety in the MPI, in wt.%.

#### 2.2.4. Gel Permeation Chromatography (GPC)

Molar mass and dispersity (*Đ*) were determined by GPC using a Polymer Laboratories GPC KF 806L, Tokyo, Japan equipped with refractive index detector and SHODEX column, Tokyo, Japan. THF was used as an eluent (1.0 mL/min) and PMMA as standards.

#### 2.2.5. Photolysis of MPI

Photolysis of MPI was conducted using a low-pressure mercury lamp (TNN 15/32, “Hanau” GmbH, Hanau, Germany), emitting predominantly at 254 nm; the applied light intensity was assessed at the sample surface. The light intensity at the sample surface was approximately 10 mW·cm^−2^ (measured at 254 nm using a calibrated UV radiometer). The distance between the lamp and the reaction vessel was maintained at 9 cm to ensure reproducible irradiation conditions. These parameters are consistent with the absorption characteristics of the pendant benzoin methyl ether derivative moieties and ensure efficient photolysis under the selected experimental conditions.

#### 2.2.6. Thermogravimetric Analyses (TGA)

The weight loss of dried sample was recorded on TGA, Mettler Toledo TGA/SDTA 851e, under nitrogen atmosphere with a constant flow rate of 100 mL min^−1^ for the entire range of experiments. The experiments were carried out at a heating rate of 5 °C min^−1^ from 25 °C to 700 °C.

#### 2.2.7. Differential Scanning Calorimetry (DSC)

DSC was performed using a Mettler Toledo DSC 821e differential scanning calorimeter under nitrogen atmosphere. The DSC samples of 1.5–3.0 mg were first cooled down to 0 °C and then heated up to 300 °C at a heating rate of 10 °C min^−1^.

#### 2.2.8. Dynamic Light Scattering (DLS)

The hydrodynamic diameter distributions of the virgin TiO_2_ and TiO_2_ nanoparticles modified with MPI were measured in EtOH suspensions using MalvernNano ZS zeta sizer. The 1.5 mg of nanoparticle samples were suspended in 2 mL of distilled EtOH and sonicated before measurement for 20 min. The measurements were therefore performed immediately after ultrasonication to ensure analysis of the dispersed fraction.

#### 2.2.9. Scanning Electron Microscope (SEM)

Electron microscopic studies were carried out using a Selmi REM106I scanning electron microscope (SEM) to investigate the morphology and surface structure of TiO_2_ particles before and after modification with MPI.

For sample preparation, a dilute suspension of TiO_2_ (unmodified or MPI-modified) in a suitable volatile solvent was first prepared and ultrasonicated to ensure uniform dispersion. A drop of the suspension was then deposited onto a conductive substrate (aluminum stub with carbon adhesive tape) and allowed to dry under ambient conditions, enabling solvent evaporation and particle immobilization on the surface.

To prevent charging effects during SEM imaging, the dried samples were coated with a thin conductive layer of copper using a sputter coating technique.

SEM images were recorded at different magnifications to assess both the agglomeration behavior and surface morphology of the particles. Measurements were performed under high vacuum conditions with an appropriate accelerating voltage to ensure optimal image quality.

### 2.3. Adsorption of MPI on Surface of Inorganic Particles

Adsorption of MPIs on the surface of inorganic particles TiO_2_ was investigated in EtAc and MeOH solutions at various MPI concentrations (from 0.1 to 8 wt.%) and different adsorption time from 1 to 120 min under continuous stirring at room temperature. The ratio of the adsorbent to solution was kept constant as 1 to 5 (by weight). To be precise, the process was performed as follows: 2 g of TiO_2_ particles were added to 10 g of MPI solution and continuously stirred using a magnetic stirrer. After adsorption process, the samples were centrifuged for 10 min at 9000 rpm to separate out the inorganic filler particles with adsorbed MPI from a solution containing unadsorbed MPI. The solid particles were dispersed in EtAc or MeOH and centrifuged once again. The amounts of MPI adsorbed on the particles were estimated using thermogravimetric analysis.

The optimal conditions for MPI adsorption on TiO_2_ filler were found to be: time—60 min at MPI concentration in EA solution—2 wt.%.

### 2.4. MMA Polymerization UV-Induced by Surface-Immobilized MPI on TiO_2_ Particles

For the investigation of UV-induced graft polymerization of MMA, the following experiment was performed. Initially, 0.1 g of MMA (corresponding to 1 mmol) was dissolved in 1 mL of ethyl acetate under stirring for 5 min. Then, 0.2 g of TiO_2_ particles functionalized with MPI was added to this solution, and the mixture was stirred for 30 min to achieve homogeneous dispersion. The experiment was similarly performed with the virgin TiO_2_ particles. The reaction mixture was irradiated using a low-pressure mercury UV lamp (TNN 15/32, Hanau GmbH, Germany). The obtained suspension was then centrifuged at 9000 rpm for 10 min to separate the polymerized material. The supernatant containing unreacted monomer and unbound PMA was discarded, and the precipitate was thoroughly washed with isopropanol and distilled water. The purified sample was centrifuged again under the same conditions to ensure complete removal of residual species. Finally, the resulting composite particles were dried under vacuum and subjected to TGA and DLS measurements.

## 3. Results and Discussion

The novel MPI was synthesized by tethering 3-hydroxy-2-methoxy-1,2-diphenylpropan-1-one (PI) moieties to the p(MMA-co-MA) macromolecules using a polymer modification reaction. The resulting MPI was characterized using IR, UV, and NMR spectroscopy, as well as DSC and TGA. The photolysis and thermolysis behavior were thoroughly investigated (Section 3.1). Detailed studies were also conducted on the functionalization of TiO_2_ with MPI (Section 3.2). The synthesized MPI exhibited high surface activity, strong adsorptive capacity, and excellent performance as a radical polymerization initiator for the fabrication of TiO_2_-based nanocomposites. Finally, UV-induced polymerization of methyl methacrylate (MMA) using MPI-functionalized TiO_2_ as a photoinitiator was successfully demonstrated (Section 3.3).

### 3.1. Synthesis and Properties of MPI

#### 3.1.1. Synthesis of MPI

The MPI was synthesized in a two-stage process. At the first stage, a copolymer p(MMA-co-MA), served further as a polymer carrier, was synthesized via conventional radical copolymerization of MMA with MA. At the second stage, the tethering of the benzoin derivative, namely 3-hydroxy-2-methoxy-1,2-diphenylpropan-1-one (PI), to this polymer carrier was achieved by acylating the hydroxyl groups of PI with anhydride groups of MA units in the macromolecular chains of p(MMA-co-MA), resulting in MPI (see Scheme 1).

The structure of the MPI synthesized was confirmed by FTIR, UV–Vis, and ^1^H NMR spectroscopies.

**Figure 1:**
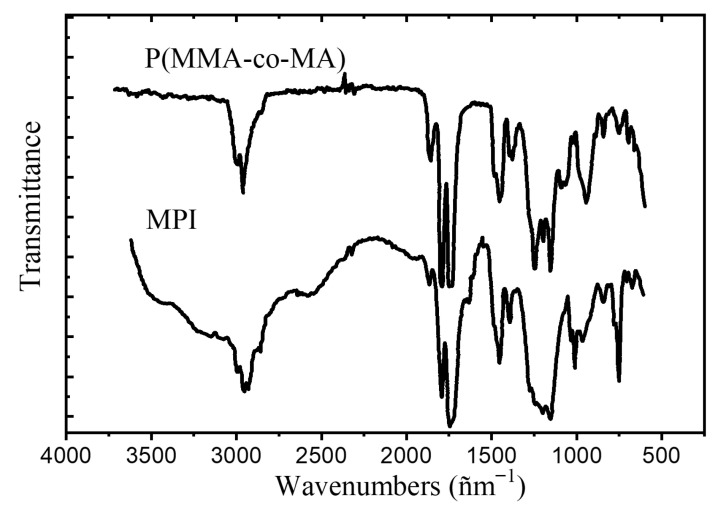
FTIR spectra of p(MMA-co-MA) and MPI-4 recorded as thin films on KBr plates.

The FTIR spectra of the initial p(MMA-co-MA) copolymer and the resulting MPI-4 are presented in Figure 1.

The FTIR spectra of p(MMA-co-MA) and the corresponding MPI reveal clear differences associated with the chemical modification of the copolymer during photoinitiator tethering. In the spectrum of p(MMA-co-MA), the dominant features include the strong ester C=O stretching band of MMA units at ~1736 cm^−1^ and the characteristic doublet of cyclic anhydride groups at ~1850 and ~1780 cm^−1^, assigned to the asymmetric and symmetric C=O stretching vibrations of the MA-derived anhydride rings. The absence of a broad O–H stretching band in the 3000–3500 cm^−1^ region and the lack of a distinct carboxylate ν_as/ν_s(COO^−^) doublet indicate that the MA units are predominantly present in the anhydride form, with negligible hydrolysis to carboxylic acid or carboxylate species.

In contrast, the FTIR spectrum of MPI-4 exhibits significant changes in the carbonyl region. The intensity of the anhydride bands decreases markedly, indicating partial ring opening of the maleic anhydride groups during the acylation reaction with the PI. The carbonyl region becomes broader, reflecting overlapping contributions from ester C=O groups, the aromatic ketone C=O of the PI fragment, and newly formed carboxylic acid (–COOH) groups, which typically absorb in the 1700–1725 cm^−1^ region. The appearance of a broad O–H stretching band in the 3300–3000 cm^−1^ region further confirms the formation of hydrogen-bonded –COOH groups in the monomaleate structures.

Importantly, neither spectrum shows a clearly defined carboxylate (–COO^−^) doublet in the 1550–1610 and 1360–1420 cm^−1^ regions, indicating that under the applied synthesis and measurement conditions the carboxyl functionalities are predominantly present in the protonated –COOH form rather than as ionic carboxylates. Additionally, the MPI spectrum displays aromatic skeletal vibrations in the 1600–1500 cm^−1^ region, confirming successful incorporation of the photoinitiator moieties into the copolymer backbone.

Overall, the comparison demonstrates that PI tethering leads to partial anhydride ring opening and formation of carboxylic acid groups, while no substantial conversion to carboxylate species is observed.

**Figure 2:**
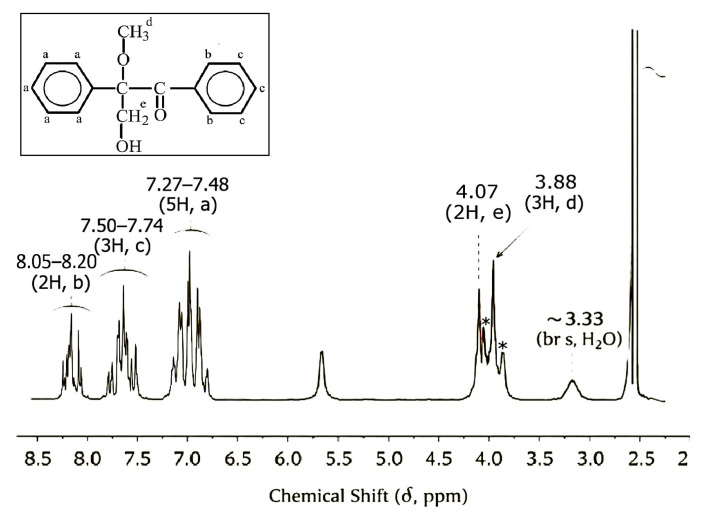
^1^H-NMR spectrum of PI.

The polymer modification reactions occurring throughout the process of tethering the PI moieties to p(MMA-co-MA) copolymer were confirmed by ^1^H NMR spectroscopy, as shown in Figure 2. Comparing the spectra of MPI-4 with those of the used intermediate compounds, namely PI and p(MMA-co-MA), demonstrated their transition to the finally functionalized copolymer with pendant aromatic moieties.

The ^1^H NMR spectrum (400 MHz, DMSO-d_6_) of PI (Figure 2) exhibits characteristic signals consistent with its molecular structure. The complex multiplets in the region δ 7.20–8.20 ppm is typically attributed to ortho, meta, and para protons of the two phenyl rings (10H). The hydroxymethyl group (–CH_2_OH) is observed as an AB-type signal centered at δ ~4.07 ppm (2H), reflecting the diastereotopic nature of the methylene protons. The methoxy group (–OCH_3_) gives a singlet at δ 3.88 ppm (3H).

Residual solvent signals of DMSO-d_6_ (δ 2.50 ppm) and water (δ ~3.33 ppm) are also present. A broad singlet of the hydroxyl proton (1H, br s, OH) is expected to be in the region ~2.0 ppm, in this case overlapping with the stronger signal of the proton in residual solvent.

**Figure 3:**
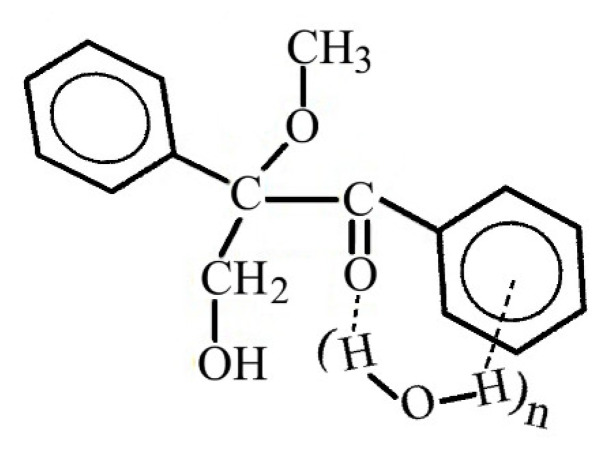
The cyclic structure of PI-(H_2_O)_n_ clusters, formed due to the formation of OH⋅⋅⋅π bonds between water molecules and the PI aromatic rings.

Some more signals in the region of ~4.0 ppm (pointed by asterisks) are most likely attributed to the protons of water molecules bonded by H-bonds with PI, as shown below (Figure 3):

The formation of water clusters with aromatic compounds, particularly with benzophenone, has been observed and described by other authors [48,49]. Such clusters, e.g., (Ph_2_C=O)–(H_2_O)_n_, are characterized by hydrogen bonding to the carbonyl oxygen and π-stacking interactions. Hydrogen bonding causes the water signal to shift downfield in ^1^H NMR, as a result, additional weak signals appear in the region ~4.0 ppm.

The formation of H-bonds between water and PI molecules explains the fact that there are some difficulties in removing water during the drying of the PI synthesized. While traces of water in PI do not have a crucial effect on the successive synthesis of MPI.

A broad signal corresponding to the hydroxyl proton (–OH) may be observed in the region δ ~5.0–5.6 ppm, depending on hydrogen bonding and exchange conditions. The residual solvent signals of DMSO-d_6_ (δ 2.50 ppm) and water (δ ~3.33 ppm) are also present. Overall, the observed chemical shifts and signal patterns confirm the successful synthesis of the hydroxyl-containing benzoin methyl ether derivative.

The ^1^H NMR spectra (400 MHz, DMSO-d_6_) of the copolymer p(MMA-co-MA) and the final product MPI are not shown due to the presence of residual solvents and limited spectral quality. Nevertheless, the obtained data clearly demonstrate the structural differences between these materials.

The copolymer p(MMA-co-MA) exhibits broad signals characteristic of polymeric systems: the methyl groups of MMA units (–CH_3_) appear as a broad signal at δ 0.85–1.20 ppm; the methylene protons of the polymer backbone (–CH_2_–) are observed as a multiplet at δ ~1.80 ppm; the methoxy groups (–OCH_3_) give signals at δ ~3.60 ppm; and the methine protons adjacent to anhydride groups (–CH–COO–) appear at δ ~3.30 ppm. In comparison, the ^1^H NMR spectrum of MPI, i.e., the polymer functionalized with aromatic photoactive moieties, shows additional signals in the aromatic region at δ 6.80–7.80 ppm, corresponding to phenyl protons and confirming the incorporation of benzoin-derived units. At the same time, characteristic signals of the polymer backbone are retained, including the methyl groups (δ 0.85–1.20 ppm) and methine protons (δ ~3.20–3.30 ppm). The methylene (δ ~1.60–1.80 ppm) and methoxy (δ ~3.60 ppm) signals become broader and more intense due to partial overlap with protons from the grafted PI fragments.

Both p(MMA-co-MA) and MPI spectra exhibit significant signal broadening compared to the sharp resonances observed for the low-molecular-weight PI, which is consistent with their macromolecular nature.

Overall, the appearance of new aromatic signals together with the preservation of characteristic polymer backbone resonances confirms the successful functionalization of p(MMA-co-MA) with aromatic side groups.

**Figure 4:**
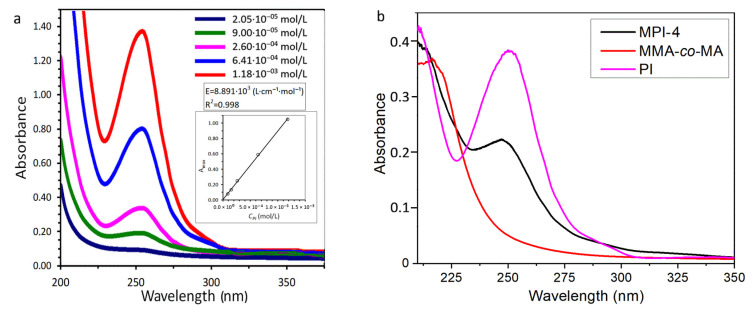
Results of UV–Vis spectroscopy: (**a**) UV-spectra of PI at various concentrations, the insert presents the plot of extinction at 252 nm vs. the PI concentration used for calculating the molar extinction coefficient; (**b**) UV-spectra of PI (C = 0.1 wt.%), MPI-4 (C = 0.05 wt.%) and p(MMA-co-MA) (C = 0.5 wt.%). All spectra were recorded in ethanol in 1 mm path quartz cuvettes, using a Shimadzu UV–1650 PC spectrometer, Shimadzu Corp., Tokyo, Japan.

Figure 4 presents the UV absorption spectra of MPI and its low-molecular-weight counterpart in ethanol. The spectra provide strong evidence for the presence of tethered photoinitiator (PI) fragments within the MPI macromolecules. Both PI and MPI exhibit similar absorption maxima at λ_max_ = 250–254 nm, characteristic of π→π* electronic transitions in aromatic ketones. In contrast, the original p(MMA-co-MA) copolymer shows no significant absorption in this region, confirming the absence of aromatic chromophores.

The extinction coefficient ε_252nm_ = 8.89 × 10^3^ L·cm^−1^·mol^−1^ was taken for calculating the content of the PI moieties in the structure of MPI. Based on these calculations, the content of tethered PI units in MPI was of 11.8 wt.%, that corresponded to the molar fraction of PI moieties equaled to 0.0438, or approximately one-tenth of the maleic anhydride units in the original p(MMA-co-MA) participating in the acylation reaction with PI. Considering that the initial molar ratio of PI:MA was 1:10, this result suggested that the reaction of PI with MA units proceeded with nearly quantitative yield.

In our previous study [43,44,45], we demonstrated that the optimal reaction time for synthesizing macrophotoinitiators bearing benzoin moieties is determined by the balance between two competing processes: (i) the covalent attachment of benzoin fragments and (ii) the thermal decomposition of benzoin units within the MPI structure. A similar time-dependent behavior was anticipated in the synthesis of MPI described here.

To estimate the optimal reaction time for the acylation reaction of PI with p(MMA-co-MA), 4 and 8 h of reaction time were examined. As shown in Table 1 below, the number-average molecular weight (*M*_n_) of MPI-4 after 4 h of reaction was significantly higher compared with *M*_n_ of the initial p(MMA-co-MA), and its value corresponded well to the theoretical calculation. This rise in *M*_n_ was obviously connected with tethering the bulky moieties of PI (*M*_w_ = 256 Da) to macromolecules of p(MMA-co-MA). However, after 8 h of reaction, the molecular weight of the obtained product was essentially reduced compared with that of MPI after 4 h of reaction. Moreover, the product obtained after 8 h of reaction had *M*_n_ even lower than for the initial p(MMA-co-MA). A possible reason for this phenomenon is the thermal decomposition of the tethered PI moieties in MPI macromolecules; and the loss of bulky PI moieties brings about a lowering of the molecular weight of MPI. The observed dropping down of the PI moieties content in MPI-8 compared with MPI-4 or MPI-6 supported this comprehension conception. The thermal decomposition of MPI-4 was investigated, and its result will be considered in the next Section 3.1.2. The free radicals formed in this process provoked breaking reactions of the macromolecule backbone, which manifested as *M*_n_ lowering. The performed study indicated that 4 h was the optimal reaction time for MPI synthesis.

Interestingly, the esterification of p(MMA-co-MA) with PI, having a primary alcohol group, proceeded more efficiently and rapidly than with benzoin [43,44,45], having a secondary alcohol group, likely because of a higher reactivity of primary alcohols toward organic acid anhydrides.

#### 3.1.2. The Photolysis and Thermal Decomposition of MPI

Photolysis is a chemical reaction in which chemical compounds are broken down as a result of photon absorption.

**Figure 5:**
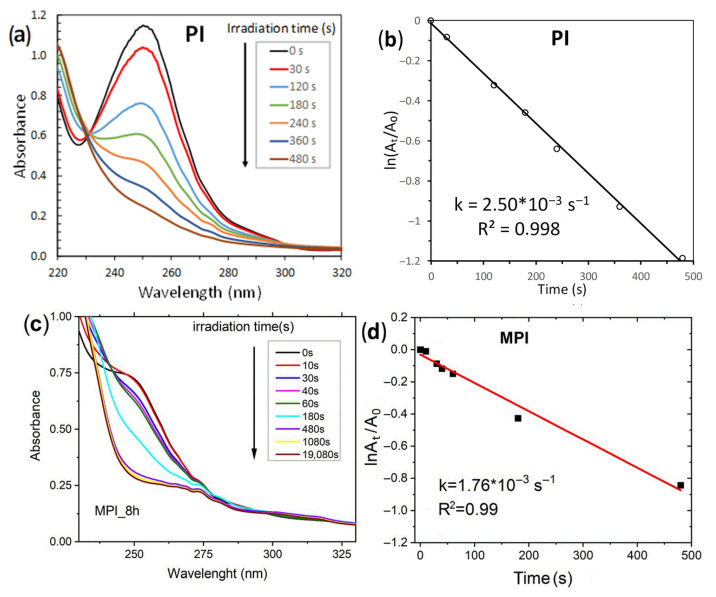
UV–Vis spectra of PI (0.02 wt.%) (**a**) and MPI-8 (0.10 wt.%) (**c**) recorded during UV irradiation, showing the decrease in absorbance due to photolysis, together with the corresponding kinetic plots of ln(A_t_/A_0_) versus irradiation time for PI (**b**) and MPI-8 (**d**). R* denotes the correlation coefficient of the linear fit.

Photolysis of PI and MPI-8 was performed in ethanol solution by a spot-light curing system in the UV-cuvette with a rubber plug. Figure 5 illustrates the UV-induced photodegradation behavior of the PI and the corresponding MPI, observed using UV–Vis spectroscopy and analyzed by kinetic modeling.

Figure 5a shows the evolution of the UV absorption spectra of the PI over the irradiation period ranging from 0 to 900 s. A strong absorption band attributed to π → π* transitions in the aromatic ketone structure of the PI was initially observed at around 250–275 nm. As the irradiation time increased, a gradual decrease in absorbance was observed, indicating the progressive photodegradation of the PI chromophore. This process was quantitatively analyzed in Figure 5b, representing a semi-logarithmic dependence (ln(A_t_/A_0_)) of the ratio of absorbance values of PI solution at time *t* (A_t_) to the initial absorbance of this solution (A_0_) plotted vs. irradiation time t. The resulting linear relationship (R = 0.99) confirmed that the degradation of the PI followed the first-order kinetics. The rate constant for this process was found to be k = 2.50 × 10^−3^ s^−1^.

Figure 5c presents the changes occurred in the UV absorption spectra of the MPI-8 sample as the irradiation time increased. MPI-8 was used in photodecomposition studies due to its benzoin content, enabling clearer and cozy monitoring of photolysis kinetics. Similarly to PI, this plot exhibits a decrease in the absorbance values of MPI in the 250–275 nm range over time. The kinetic analysis of MPI degradation is shown in Figure 5d. The ln(A_t_/A_0_) values also display a linear relationship with irradiation time (R* = 0.99), confirming that the process obeyed the first-order kinetics. The calculated rate constant for MPI was k = 1.76 × 10^−3^ s^−1^, which is about 25% lower than that of the unbonded PI. The kinetic analysis was performed within the irradiation time range where a linear relationship between ln(A_t_/A_0_) and time was observed, corresponding to apparent first-order behavior. It should be noted that, in preliminary experiments, a significantly broader irradiation time range was investigated. However, at longer irradiation times (high conversion degrees), deviations from linearity became evident, likely due to secondary processes such as radical recombination and depletion of chromophores. Therefore, the analysis was intentionally restricted to the linear region to ensure reliable determination of the rate constant. Slight deviations at extended irradiation times (e.g., 480 s for MPI) may occur and do not affect the overall kinetic interpretation, as the rate constants are primarily determined from the initial linear regime.

Both PI and MPI degrade under UV light following first-order kinetics, but MPI demonstrates slower degradation caused by UV light irradiation. Let us consider the reasons for a lower rate constant of photolysis, i.e., the increased photostability of MPI compared to unbonded PI.

Bonding of chromophore molecules to polymer chains brings about an increase in their photostability, while the chromophore electron structure is not changed—no shift in λ_max_ is observed (Figure 4). Some papers [33,50] have also reported on a decrease in the photolysis rate and the photoinitiation activity of MPIs compared with that of its low-molecular counterpart.

To explain this phenomenon, it is worth considering the evolution of the excited states of PI after light absorption that can be represented by the scheme [4]:

$$\mathrm{PI}(S_{0})\overset{1h\nu }{\to }\mathrm{PI}(S_{1})\overset{\mathrm{ISC}}{\to }\mathrm{PI}(T_{1})\to {\overset{•}{R}}_{1}+{\overset{•}{R}}_{2}\cdot (R3)$$

where a singlet state PI(*S*_1_) is formed after the photon absorption by molecules of PI(*S*_0_) in the ground state; afterward, PI(*S*_1_) can transform via intersystem crossing (*ISC*) into PI(*T*_1_) triplet state, which can further undergo a cleavage reaction with formation of free radicals.

Alternatively, PI(*S*_1_) can undergo a photon splitting (photoluminescence) or deactivation owing to the energy dissipation through atom vibration and molecular motion. On the other hand, the PI(*T*_1_) triplet state can be deactivated owing to the scavenge mechanism at interaction with other molecules (scavengers). Both these processes obviously lead to slowing down the decomposition rate of PI. It can be supposed that carrier macromolecules (MMA-MA) may act as a scavenger, but this is not realized in fact. Similar results have been reported in other investigations; e.g., some authors [51,52] investigated photolysis of low-molecular and polymeric PI and established that macromolecules of polymer have a negligible triplet quenching effect. Thus, it can be concluded that the macromolecules of the polymer carrier (owing to the multiple atoms and chemical bonds in its structure) rather dissipate energy from the excited PI(*S*_1_) state, leading to its deactivation and consequently lowering the photolysis rate of PI moieties when bonded to the polymer carrier.

The photolysis mechanism of benzoin derivatives has been described in detail in several studies [53,54]. Therefore, it can be assumed that the photolysis of the low-molecular photoinitiator (PI) and its high-molecular analog (MPI) investigated in this work proceeds via a similar mechanism.

**Scheme 3:**
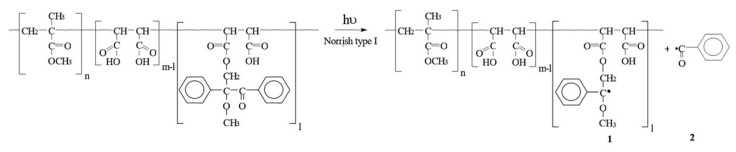
The scheme of MPI photolysis.

Benzoin and its derivatives are among the most widely used photoinitiators in photopolymerization due to their high quantum efficiency and the high reactivity of the generated free-radical species. Previous studies have shown that their photolysis is dominated by a Norrish type I cleavage, which represents the primary process and results in the formation of a macroradical (1) and a low-molecular-weight radical (2). Accordingly, the photolysis of the synthesized MPI can be described by Scheme 3.

The subsequent reactions of these radicals depend on the medium in which photodegradation occurs. In the presence of vinyl monomers, they can initiate photopolymerization and promote the curing of unsaturated resins and compositions. Through hydrogen abstraction followed by recombination reactions, they may also induce crosslinking in various polymers. In organic solvents, the radicals undergo fragmentation, hydrogen abstraction, and recombination reactions, leading to the formation of various products, predominantly benzil, acetophenone, pinacol ether derivatives, and benzaldehyde.

**Figure 6:**
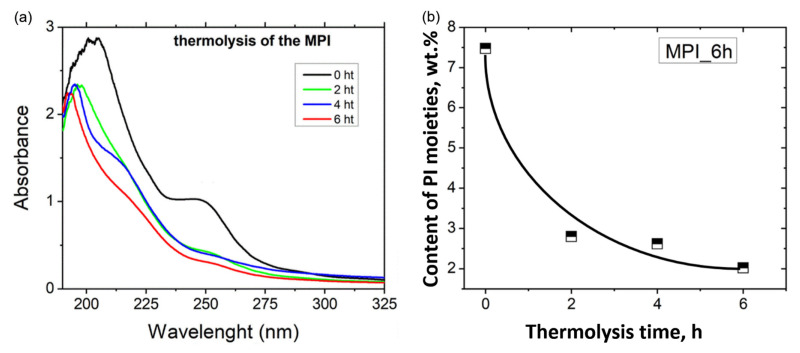
(**a**) UV-spectra of the MPI-6 after thermal decomposition at T = 130 °C for various time; (**b**) the content of PI moieties in MPI-6 vs. decomposition time at T = 130 °C.

Figure 6 represents the results of thermal decomposition (thermolysis) of MPI-6 at 130 °C, assessed via UV spectroscopy and the content of PI moieties. MPI-6 was selected for thermal decomposition experiments because it contains an optimal amount of benzoin groups, allowing reliable evaluation of thermal stability without excessive overlap of degradation processes associated with high photoinitiator loading. The UV absorption spectra (Figure 6a) of the MPI samples after different thermolysis durations (0, 2, 4, and 6 h) illustrate an essential decrease in absorption with increasing thermolysis time, which is apparently caused by decomposition of the PI fragments in MPI. At 0 h, the spectrum showed a clearly distinct characteristic peak at 250 nm. As the thermolysis progressed, the intensity of this peak systematically decreased, indicating gradual degradation or detachment of the PI fragments from the MPI matrix. By 6 h, the absorbance is significantly reduced across the spectrum, suggesting extensive decomposition or removal of the chromophores. The content of PI moieties in the MPI structure as a function of thermolysis time shows a clear downward trend (Figure 6b). Initially, the content of PI moieties was around 7.5 wt.%, confirming their significant presence in the unheated sample. After 2 h of thermal treatment, the PI moieties content dropped sharply to approximately 3 wt.%, and further decreased to about 2 wt.% after 6 h. This behavior confirms a cleavage and loss of the chromophore groups in the MPI structure because of their thermal degradation and explains the existence of an optimal time for MPI synthesis. Thermolysis of benzoin was not still studied, while in paper [55], the authors successfully applied this process for the synthesis of copper nanowires. They stated that benzoin thermolysis occurred via radical mechanism, and the free radicals formed can play a role of a reducing agent when interacting with Cu^2+^ ions.

#### 3.1.3. Thermal Analyses of the P(MMA-co-MA) and MPI (TGA and DSC)

TGA analysis was used to study p(MMA-co-MA) and MPI-4 degradation. To have a comparable structure of both MPI-4 and p(MMA-co-MA), the latter has been hydrolyzed via precipitation in water to transform into acid form.

**Figure 7:**
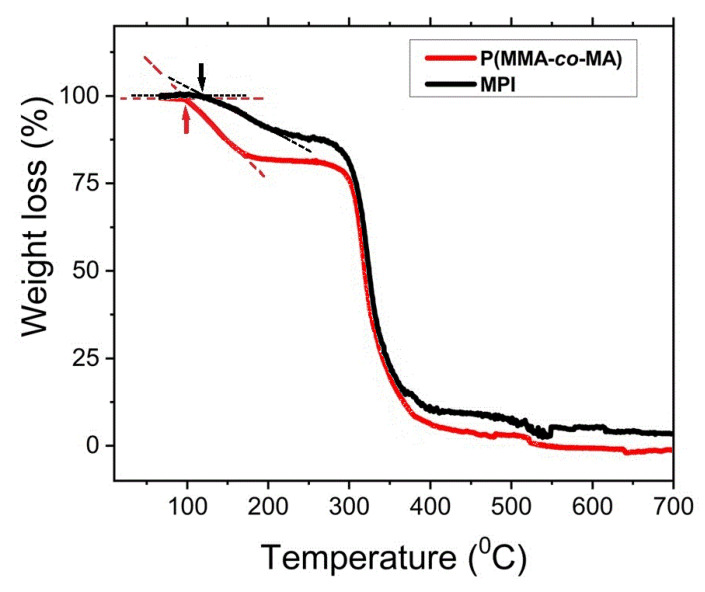
TGA analysis for copolymer P(MMA-co-MA) and MPI-4. The arrows point to the T_onset_ on the thermograms, determined as the intersection points of the tangent lines (dashed lines).

TGA measurements were performed under a nitrogen atmosphere to prevent oxidative degradation and to evaluate the intrinsic thermal stability of the materials. The inert nitrogen environment eliminates oxygen-induced reactions, ensuring that the recorded mass loss corresponds solely to thermal decomposition processes rather than oxidative combustion. The TGA diagrams of P(MMA-co-MA) and MPI-4 (Figure 7) demonstrate their quite similar behaviors, although MPI is more stable to weight loss up to 290 °C.

**Scheme 4:**
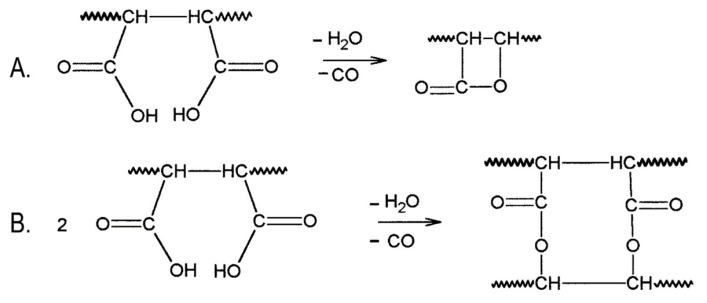
Degradation of maleic acid copolymers via intramolecular (**A**) and intermolecular interactions (**B**).

The TGA thermograms of both P(MMA-co-MA) and MPI-4 show two-stage degradation. The first stage of thermal decomposition, when the samples began to lose their weight occurs in the temperature range between 110–205 °C and corresponds to around 12–18% of weight loss. When analyzing the TGA thermograms, one can notice a significant weight loss at about 100–200 °C. Similar results were described by M. Świtała-Żeliazkow [56], who found that styrene–maleic acid copolymer undergoes degradation in three stages. The first stage occurs in a temperature range of about 100–200 °C, and the weight loss is caused by the release of water (maximum at 154 °C) and carbon monoxide (maximum at 160 °C) as a result of the following reactions:

The TGA diagrams of P(MMA-co-MA) copolymer and MPI-4 (Figure 7) demonstrate quite similar behaviors, although MPI-4 is more stable to weight loss up to 290 °C. The T_onset_ of MPI-4 is shifted to a higher temperature compared to that of P(MMA-co-MA)—108 °C vs. 100 °C respectively, as well as thermodegradation of MPI proceeds at a lower rate. There are two possible reasons for this phenomenon: (i) a lower concentration of carboxylic groups in MPI macromolecules compared with P(MMA-co-MA), as these groups are partially esterified in MPI; (ii) the presence of bulky PI moieties in macromolecules of MPI can hinder intermolecular interactions resulted in release of water and carbon monoxide (Scheme 4B).

Decomposition processes were completed at decomposition temperatures between 294–383 °C, the results being evidence for the decomposition of the main polymer chains.

**Figure 8:**
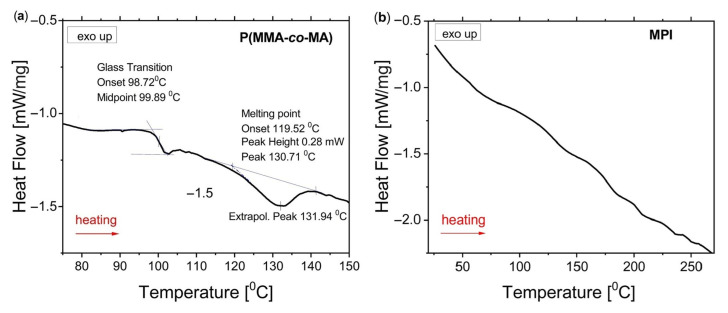
DSC thermogram for p(MMA-co-MA) (**a**) and MPI-4 (**b**).

The DSC thermograms of the p(MMA-co-MA) copolymer and MPI-4 were recorded during the heating cycle with the exothermic direction oriented upwards (Figure 8a,b). In both cases, the slight negative slopes of the DSC curves are clearly associated with the aforementioned endothermic degradation processes of the p(MMA-co-MA) copolymer and MPI-4, as described in the analysis of the TGA diagrams (Figure 7). In addition, the curve of MMA-co-MA reveals two distinct thermal transitions: a glass transition and a melting process. The glass transition is observed with an onset temperature of 99 °C and a midpoint (T_g_) of 100 °C. The melting process begins at an onset temperature of 120 °C and reaches a peak at 131 °C. The endothermic nature of this peak suggests the presence of crystalline or semi-crystalline domains within the copolymer, likely attributed to ordered sequences of methyl methacrylate units. Overall, the DSC analysis demonstrates that p(MMA-co-MA) is a semi-crystalline material, exhibiting both a well-defined glass transition and a melting peak. The presence of these two transitions supports the assumption of phase heterogeneity.

In contrast, the DSC thermogram of MPI-4 reveals no prominent thermal events associated with polymer transition processes within the observed temperature interval. The main reason is the presence of bulky pendant groups, namely pendant PI moieties. It is well known that bulky pendant groups have a strong influence on polymer transition temperatures, especially the glass transition temperature (T_g_), which increases in this case. This phenomenon is explained by: (i) restricted chain mobility, because large substituents create steric hindrance, making polymer chains harder to rotate and move; (ii) reduced segmental flexibility, because the backbone becomes effectively stiffer. Thus, we can suppose that the T_g_ of MPI is significantly higher than that of the p(MMA-co-MA) copolymer because of its more rigid and structurally constrained polymer network. Another reason is that at higher temperatures, MPI undergoes a decomposition, leading to its crosslinking (see Scheme 4). The crosslinking, in turn, brings about a further significant increase in T_g_.

### 3.2. Functionalization of TiO_2_ Particles with MPI and Study of Their Properties

There is a growing scientific and industrial interest in surface modification techniques aimed at tailoring the physical, chemical, and functional properties of materials to meet specific application needs. Surface engineering enables the introduction of desirable characteristics such as improved compatibility, enhanced mechanical performance, targeted reactivity, and added functionality, especially when dealing with composite systems or hybrid materials [57,58]. Among these techniques, photo-induced surface modification using photopolymerizable systems has attracted significant attention [59,60]. One particularly promising strategy involves the use of MPIs, which initiate polymerization upon light exposure. In recent research, MPI have been synthesized to contain polyfunctional structures with highly reactive polar groups such as carboxylic acids and anhydrides. These functional groups not only contribute to photoinitiation but also enhance chemical interactions with the substrate surface, thereby making MPI effective agents for modifying inorganic filler materials.

The immobilization of MPI onto the surface of mineral fillers has been investigated as a means of improving filler dispersion and interfacial bonding within polymer matrices. In particular, rutile-phase titanium dioxide (TiO_2_) has been selected as a model filler for surface modification studies. TiO_2_ is widely used due to its multifunctional properties, it serves as a white pigment, UV-blocker, photocatalyst, and an inert filler in polymer composites. Additionally, it has notable applications in biomedical devices, especially metallic implants, owing to its biocompatibility and corrosion resistance [61,62,63].

Adsorption of both p(MMA-MA) and MPI-4 onto the TiO_2_ surface proceeds through a multistep interfacial mechanism involving hydrogen bonding, surface-assisted chemical transformation, and coordination interactions.

At the initial stage, protonated carboxylic acid groups (–COOH), either formed by partial hydrolysis of maleic anhydride units or already present in the polymer structure, may interact with surface hydroxyl groups (Ti–OH) via hydrogen bonding [64]. In addition, the carbonyl groups of cyclic maleic anhydride can participate in weak donor–acceptor interactions with Lewis-acidic Ti^4+^ surface sites. These interactions are relatively weak and reversible but facilitate close approach of the macromolecule to the oxide surface.

Upon contact with the hydroxylated TiO_2_ surface, maleic anhydride units can undergo surface-assisted ring opening [65]. Nucleophilic attack by surface Ti–OH groups leads to cleavage of the anhydride ring, resulting in the formation of carboxylate species capable of coordinating to Ti^4+^ centers, together with additional free carboxylic acid groups along the polymer chain. This transformation effectively converts relatively weakly interacting anhydride functionalities into strongly anchoring carboxylate groups.

Subsequently, deprotonation at the interface leads to the formation of coordinated carboxylate (–COO^−^) species, which bind to TiO_2_ through inner-sphere complexation. Such coordination interactions are significantly stronger than hydrogen bonding and represent the dominant anchoring mechanism for both polymers.

In contrast to low-molecular carboxylic acids [66], polymeric systems such as p(MMA-MA) and MPI provide multiple functional groups along the macromolecular chain, enabling multivalent surface attachment. This cooperative binding increases adsorption strength, reduces desorption probability, and promotes the formation of a stable interfacial layer. In the case of MPI, the presence of pre-formed monomaleate structures and additional polar functionalities further enhances the density of potential anchoring sites and contributes to improved stabilization of the modified TiO_2_ particles. Notably, this adsorption mechanism is conceptually analogous to that of commercial dispersants such as DISPERBYK-2080 (a polyether-modified styrene–maleic anhydride copolymer), which also relies on anhydride-derived carboxylate anchoring groups for oxide surface modification, although the molecular architecture and functional density of p(MMA-MA) and MPI differ from that of the commercial benchmark.

Overall, adsorption of both p(MMA-MA) and MPI onto TiO_2_ is governed by a combination of initial hydrogen bonding, surface-induced anhydride ring opening, formation of coordinated carboxylate species, and multivalent polymer–surface interactions, leading to stable surface modification and enhanced colloidal stability.

A similar concept was explored by Luzinov et al. [67], where a copolymer of styrene and maleic anhydride containing peroxy structures was immobilized onto particle surfaces to investigate photochemical modification. The anhydride functionalities played a critical role in adsorption-based immobilization the polymer to the filler, offering a precedent for employing multifunctional MPI in surface treatments. Overall, the surface functionalization of rutile TiO_2_ using macromolecular photoinitiators holds potential for developing advanced composites with improved dispersion, reactivity, and performance.

#### 3.2.1. Study of the Immobilization and TGA

**Figure 9:**
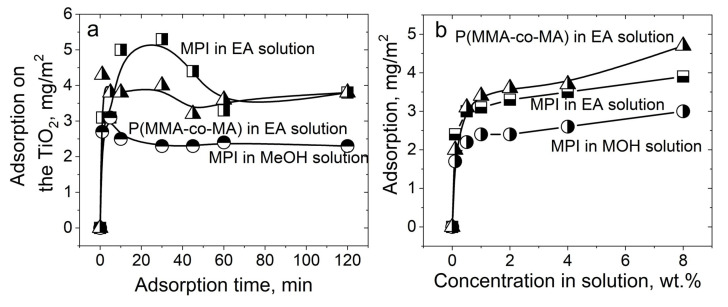
(**a**) Adsorption kinetics of MPI-4 and p(MMA-co-MA) from organic solutions (2 wt.%) at the surface of dispersed TiO_2_; (**b**) adsorption isotherms of MPI-4 and p(MMA-co-MA) at the surface of dispersed TiO_2_ from organic solutions (adsorption time: 60 min).

Immobilization of MPI-4 at the surface of TiO_2_ fillers was achieved through its adsorption from EtAc or MeOH solutions. Figure 9 illustrates the main features of MPI-4 adsorption at the surfaces of TiO_2_. The amount of adsorbed MPI depended on the adsorption time, solvent, initial MPI concentration in the solution, and the filler nature.

Adsorption of MPI-4 at the surface of inorganic particles TiO_2_ was investigated in EA and MeOH solutions at MPI-4 concentration 2 wt.% and various adsorption times (5 to 60 min), under continuous stirring at room temperature. Ratio of the adsorbent to solution was kept constant as 1 to 5. After adsorption the samples were centrifuged for 10 min (9000 rpm), the unbounded amount of MPI was removed by washing in solvent out and the rest was centrifuged once again. The amount of MPI adsorbed onto particles was estimated using thermogravimetric analysis. The TiO_2_ surface contains Lewis acid and base centers, which determine its adsorption and catalytic properties and define the specificity of strategies for its modification. The kinetic curves of copolymer adsorption on TiO_2_ (Figure 9a) exhibit a complex profile that can be divided into several stages. Initially, there is a sharp rise in adsorption, followed by an adsorption maximum and then a final plateau. These transitions can be attributed to changes in the structure of the adsorbed layer. The initial rapid adsorption stage indicates a high affinity of the copolymer macromolecules for the TiO_2_ surface, due to a high adsorption constant or a significant predominance of the adsorption rate over the desorption rate. The pronounced maximum observed in the kinetic curve reflects conformational transformations within the adsorbed layer. At the fast adsorption stage, macromolecules adsorb without significant conformational rearrangements, forming loose layers. As adsorption proceeds, these layers become more compact, corresponding to the observed maxima. Subsequent conformational changes lead to a rearrangement of macromolecules into flatter conformations, with active sites oriented toward the surface, which enhances their attachment. This process involves partial desorption, resulting in a slow approach to adsorption–desorption equilibrium. A similar scenario of MA copolymer adsorption on the mineral surface was described in the paper [68]. In general, the adsorption behavior of MPI-4 and p(MMA-co-MA) from AC is similar. In contrast, adsorption of MPI-4 from MeOH solution onto TiO_2_ exhibits distinctly different behavior compared to adsorption from EA. MPI-4 adsorption from MeOH increases rapidly during the first few minutes; after this initial stage, the adsorption curve quickly levels off and remains nearly constant over the entire investigated time range. Importantly, no pronounced maximum is observed, unlike in the case of EA solution. This indicates that adsorption equilibrium is reached rapidly in MeOH and that no significant conformational rearrangements of the adsorbed macromolecules occur.

The adsorption isotherm for TiO_2_ (Figure 9b) further supports this mechanism. At low copolymer concentrations, a sharp increase in adsorption is observed, indicating strong initial interaction. With increasing concentration, adsorption continues to rise but with a lower slope, suggesting the gradual formation of denser adsorbed layers. High adsorption values on the TiO_2_ surface can be achieved under conditions of short adsorption times (10–20 min) and high copolymer concentrations (>5 wt.%). The benzoin ethers groups incorporated into the p(MMA-co-MA) copolymer structure had no significant effect on the adsorption characteristics. In both cases, adsorption reached a plateau at around 5 wt.% concentration after approximately 1 h, indicating optimal conditions for immobilization on TiO_2_. Adsorption of MPI-4 on the TiO_2_ surface primarily proceeds through physical adsorption mechanisms due to the relatively low chemical reactivity of TiO_2_. Nevertheless, a strong affinity of MPI-4 macromolecules toward the TiO_2_ surface enables effective immobilization. The adsorption from MeOH increases moderately with increasing MPI-4 concentration in solution. However, even at higher concentrations (up to 8 wt.%), the adsorption capacity remains lower compared to EA. The curve shows a gradual rise followed by a tendency toward saturation, suggesting limited surface coverage.

The behavior of MPI-4 in MeOH can be attributed to the higher polarity and stronger hydrogen-bonding ability of methanol. MeOH competes with the polymer functional groups for interaction with TiO_2_ surface sites and more efficiently solvates the polar moieties of MPI. Consequently, polymer–surface interactions are weaker in MeOH than in EA, resulting in lower adsorption values and the absence of a pronounced adsorption maximum. Furthermore, at room temperature and in the absence of catalytic conditions, methanol is unlikely to induce significant chemical conversion of anhydride groups. Instead, hydrogen bonding and solvation effects predominate, further weakening polymer–surface interactions and limiting adsorption.

**Figure 10:**
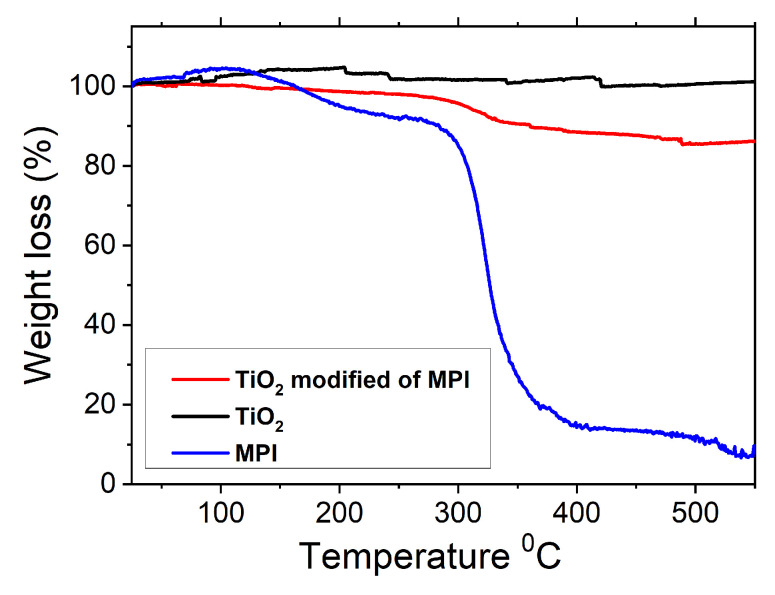
TGA curves obtained for TiO_2_, MPI-4, and MPI-4 adsorbed for 60 min on TiO_2_.

The results of the TGA analysis for TiO_2_, MPI-4, and MPI-4 adsorbed on TiO_2_ are presented in the Figure 10. A weight loss of approximately 10% for the sample with MPI-4 adsorbed on TiO_2_ indicates significant adsorption of MPI-4 onto the TiO_2_ nanoparticles. While pure TiO_2_ exhibited no notable weight loss, the MPI-4 sample showed a clear, multi-stage degradation pattern. The first stage, from 20 to 280 °C, involved a weight loss of nearly 10%, followed by a second stage between 280 and 375 °C, where about 70% of the total weight was lost. A slower degradation process, resembling the initial stage, was observed at the third stage. A similar temperature-dependent degradation behavior was noted for MPI-4 adsorbed on TiO_2_ after 60 min of adsorption, suggesting that the adsorbed polymer maintains thermal degradation characteristics close to that of the pure MPI-4.

#### 3.2.2. Dispersibility of the Modified Particles in Organic Solvents

**Figure 11:**
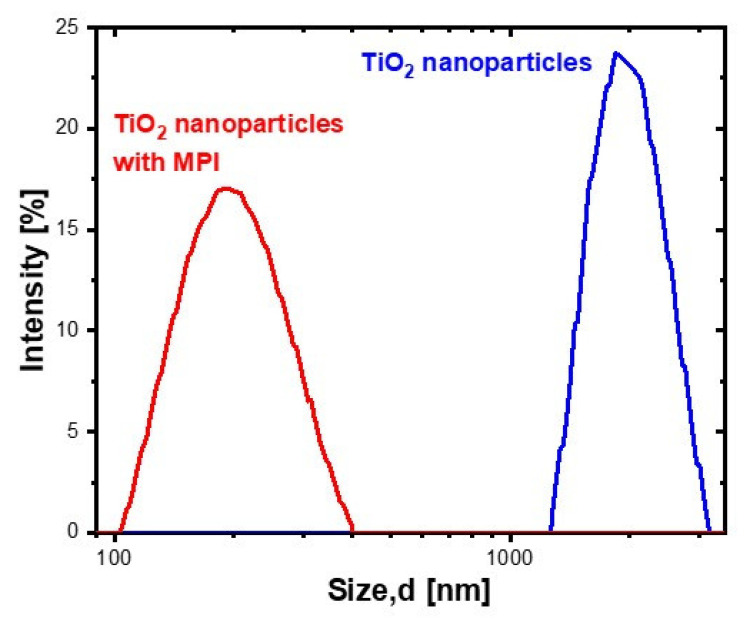
DLS analysis of virgin TiO_2_ nanoparticles and TiO_2_ nanoparticles modified with MPI-4 for 60 min.

An essential aspect of modifying inorganic nanoparticles with macromolecular MPIs is the significant enhancement of their dispersibility in organic solvents. This property is particularly important for the preparation of homogeneous polymer nanocomposites and coatings, where good dispersion of fillers determines both processing characteristics and the final properties of the material. The DLS analysis was conducted to evaluate the dispersibility of unmodified and MPI-modified TiO_2_ nanoparticles in ethyl acetate. The measurements were performed using ethanol-diluted dispersions (0.0015 g of TiO_2_ sample in 2 mL of ethanol), and the results are presented in Figure 11 and Table 2. Before surface modification, the TiO_2_ nanoparticles exhibited a Z-average hydrodynamic diameter of approximately 2822 nm, with a relatively broad size distribution (polydispersity index, PDI = 0.22), indicating significant agglomeration and poor dispersion in the medium. The particle size distribution by intensity and volume were 2524 nm and 2591 nm, respectively, confirming the presence of large aggregates in the system (Figure 11).

After modification with MPI-4, a dramatic improvement in dispersibility was observed. The Z-average size of the TiO_2_ particles decreased significantly with increasing modification time: After 5 min, the Z-average size decreased to 285.0 nm (PDI = 0.05), with a narrow and monomodal distribution. After 30 min, a Z-average of 298.7 nm was observed; however, a minor fraction of larger aggregates (~4786 nm by intensity) was also detected, indicating partial dispersion improvement. After 60 min, the best dispersibility was achieved. The Z-average was reduced to 235.9 nm (PDI = 0.15), and a uniform single-peak distribution was observed across all distribution modes: 253.5 nm (intensity), 189.2 nm (volume), and 159.0 nm (number) (Figure 11).

These results demonstrate that surface modification with MPI significantly enhances the compatibility and dispersion of TiO_2_ nanoparticles in organic solvents like ethyl acetate, reducing particle agglomeration by over tenfold. This stabilization can be explained by the adsorption mechanism of MPI. Upon contact with the hydroxylated TiO_2_ surface, maleic anhydride-derived functionalities within MPI undergo surface-assisted ring opening and/or interfacial deprotonation, forming coordinated carboxylate species that bind to surface Ti^4+^ centers. These inner-sphere coordination interactions provide strong anchoring of the macromolecule. Simultaneously, the polymeric backbone of MPI extends into the dispersion medium, introducing steric stabilization. The observed increase in the absolute value of the zeta potential (from −11.1 mV for unmodified TiO_2_ to −26.0 mV after modification) further confirms enhanced electrostatic stabilization (Table 3).

#### 3.2.3. SEM Analysis of the TiO_2_ Particles

**Figure 12:**
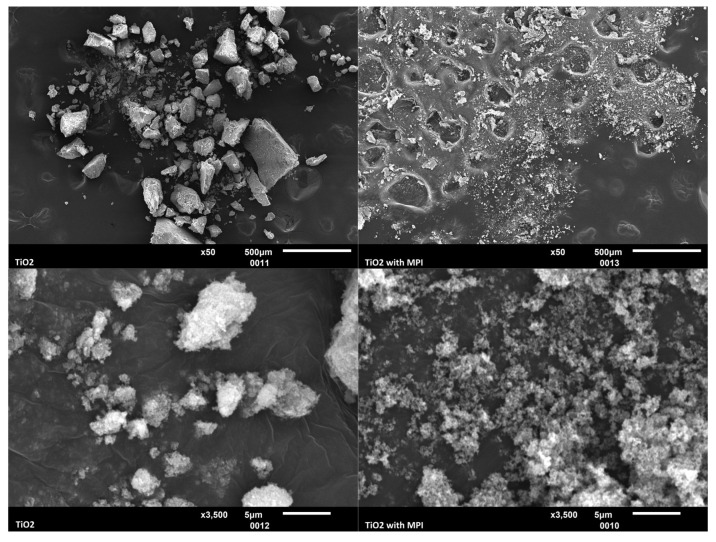
SEM images of TiO_2_ (**left**) and TiO_2_ modified with MPI-4 (**right**).

Scanning Electron Microscopy (SEM) was employed to investigate the morphology and surface structure of TiO_2_ nanoparticles before and after surface modification with MPI. Representative SEM images are presented in Figure 12. Unmodified TiO_2_ particles (Figure 12, left) exhibit extensive agglomeration, forming large, irregular clusters. This behavior is typical for untreated inorganic nanoparticles due to their high surface energy and lack of surface passivation, which promotes interparticle attractions such as van der Waals and capillary forces during drying or solvent evaporation. In contrast, TiO_2_ particles modified with MPI after 60 min of reaction time (Figure 12, right) display a substantially altered morphology. The modified particles are more uniformly distributed and appear as significantly smaller, discrete units. The suppression of agglomeration and formation of individual or weakly associated particles can be attributed to the successful surface functionalization with MPI. The MPI chains likely provide steric stabilization and increase compatibility with organic solvents, reducing particle–particle interactions and improving dispersion.

These morphological observations are in strong agreement with the DLS results (Section 3.2.2), confirming the enhanced dispersibility of MPI-modified TiO_2_ and highlighting the effectiveness of the surface modification protocol.

### 3.3. UV-Induced Polymerization of MA Using as an Initiator the TiO_2_ Particles Functionalized by MPI-4

The core concept of this study was to utilize macromolecular photoinitiators (MPIs) adsorbed on TiO_2_ nanoparticle surfaces as localized initiators of radical polymerization under UV irradiation. Specifically, the polymerization of methyl methacrylate (MMA) was carried out in aqueous media using MPI-functionalized TiO_2_ as both a carrier and a photoinitiator system. After UV exposure, the modified particles were subjected to centrifugation and multiple washing steps to remove unbound PMA. The final product was characterized by dynamic light scattering DL) to assess the growth of polymer chains on the nanoparticle surfaces. Table 3 summarizes the DLS data and Z-potential values before and after polymerization. Notably, the Z-average hydrodynamic diameter increased from 235.9 nm (MPI-modified TiO_2_) to 347.0 nm after polymerization, indicating successful grafting of poly(methyl methacrylate) (PMMA) from the surface. This increase in particle size, along with higher polydispersity index (PDI = 0.37), confirms the formation of polymer shells around the nanoparticles.

Control experiments using unmodified TiO_2_ fillers demonstrated that photopolymerization could occur without MPI, albeit at a slower rate. This is attributed to the semiconducting properties of TiO_2_ (bandgap ~3.0 eV), which allow them to act as intrinsic photoinitiators under UV exposure. TiO_2_ nanoparticles, as well as other semiconductor nanoparticles, can themselves initiate the photopolymerization of various monomers; the mechanism of this process has been considered in [69,70,71]. Upon UV irradiation, conduction-band electrons (e^−^_cb_) and valence-band holes (h^+^_vb_) are generated on the surface of semiconductor particles:

TiO_2_ + hν → *e*^−^_cb_ + *h*^+^_νb_

These reactive species can initiate radical formation through interactions with ambient substances:

*h*^+^_νb_ + ROH → RO• + H^+^

*h*^+^_νb_ + RCOO^−^→ RCOO• → R• + CO_2_

The presence of carboxyl groups in the MPI enables interfacial interactions with the TiO_2_ surface, facilitating adsorption and anchoring of the macromolecular photoinitiator. The immobilized MPI on the particle surface significantly enhances polymerization efficiency compared to systems containing unmodified (crude) TiO_2_ fillers.

Moreover, although both the crude TiO_2_ and the TiO_2_ surface-modified with MPI can initiate photopolymerization of MMA, only in the latter case is it possible to form a PMMA shell grafted to the TiO_2_ surface. When photopolymerization is initiated by the surface-immobilized MPI, the initial free radicals, formed as a result of MPI photodegradation, are tethered to the TiO_2_ surface, thereby enabling the grafting of PMMA chains. In contrast, the unmodified TiO_2_ surface promotes the formation of the initial free radicals in the bulk that are not bound to the surface, and no grafted polymer is formed.

It should be emphasized that polymerization results in a crosslinked polymer composite in which TiO_2_ particles are embedded within the cured network. The adsorbed MPI nanolayer acts as a compatibilizing interfacial modifier, promoting more uniform dispersion of inorganic fillers and reducing agglomeration during curing. In addition, the surface-bound photoinitiator generates radicals directly at the polymer–particle interface, improving interfacial adhesion and contributing to the formation of a more homogeneous crosslinked three-dimensional network.

Furthermore, immobilization of the photoinitiator moieties within the macromolecular structure minimizes issues associated with low-molecular-weight initiators, such as migration, leaching, and non-uniform distribution, which can negatively affect long-term performance and durability, particularly in coating and biomedical applications.

## 4. Conclusions

In this paper, we present a novel multifunctional photoreactive macromolecular photoinitiator (MPI) based on a copolymer of maleic anhydride and methyl methacrylate, functionalized with pendant moieties of 3-hydroxy-2-methoxy-1,2-diphenylpropan-1-one. The MPI was synthesized via a polymer-analogous reaction involving the acylation of aromatic alcohols by maleic anhydride units within the copolymer backbone. This approach enables the controlled incorporation of chromophoric groups into the polymer structure. The resulting MPI was thoroughly characterized using IR, UV, and NMR spectroscopy, along with DSC and TGA. Its photolytic and thermal decomposition behaviors were systematically investigated.

Comprehensive studies were also conducted on the functionalization of TiO_2_ particles with MPI. The synthesized MPI demonstrated high surface activity, strong adsorption capability, and excellent efficiency as a radical photoinitiator for the preparation of TiO_2_-based nanocomposites. The key aspects of polymer nanolayer formation on TiO_2_ nanoparticles surfaces are discussed in detail. It was shown that MPI immobilized on TiO_2_ particles effectively initiated the UV-induced polymerization of methyl methacrylate. The final product obtained after UV irradiation is a crosslinked polymer composite material in which TiO_2_ particles modified with MPI are uniformly embedded within the cured three-dimensional polymer network. DLS and SEM analyses confirmed successful surface modification and polymer grafting. This hybrid approach, combining surface-engineered mineral fillers with photoreactive MPI structures, offers a promising and versatile strategy for designing high-performance polymer nanocomposites.

## Figures and Tables

**Table 1:** Effect of reaction time on molecular weight and content of the tethered PI moieties in the MPI synthesized ***.

Samples	p(MMA-co-MA)	MPI-4	MPI-6	MPI-8
Reaction time, h		4	6	8
Molecular weight (*M*_n_), Da	11,500	14,300 **	11,900	9600
Dispersity *Đ*	3.86	1.65	1.96	2.09
The content of the tethered PI moieties in MPI, wt.% *	0	11.8	7.5	5.30

* Calculated from the results of UV spectroscopy. ** A theoretical value of the MPI *M*_n_ calculated according to the reaction scheme (Scheme 2) should be 13,725 Da. The deviation between the theoretical and experimental values of the MPI *M*_n_ is about 4%. *** The samples of MPIs synthesized for different times hereinafter referred to as the MPI-N, where number N corresponds to the synthesis time.

**Table 2:** Properties of the TiO_2_ and modified TiO_2_ determined from DLS.

Samples	Modification Time, min.	d (nm)				Peak No.
		Z-Average	Intensity PSD *	Volume PSD	Number PSD	
TiO_2_ unmodified	0	2822 PdI ** = 0.22	2524 (100%)	2591 (100%)	2314 (100%)	1
TiO_2_ modified	5	285.0 PdI = 0.05	304.0 (100%)	187.6 (35.9%)	210.1 (100%)	1
			0.0	323.1 (64.1%)	0.0	2
TiO_2_ modified	30	298.7 PdI = 0.22	345.6 (97.6%)	172.6 (60.3%)	153.3 (100%)	1
			4786 (2.4%)	498.5 (26.0%)	0.0	2
			0.0	4902 (13.6%)	0.0	3
TiO_2_ modified	60	235.9 PdI = 0.15	253.5 (100%)	189.2 (100%)	159 (100%)	1

* PSD—particle size distribution; ** PdI—polydispersity index.

**Table 3:** Properties of the modified TiO_2_ determined from DLS.

Sample	d (nm)			Zeta Potential (mV)
	Z-Average	Intensity PSD	Volume PSD	
TiO_2_ unmodified	2822 PdI = 0.22	2524 (100%)	2591 (100%)	−11.1
TiO_2_ MPI-4 modified for 60 min	235.9 PdI = 0.15	253.5 (100%)	189.2 (100%)	−26.0
TiO_2_ with PMMA-grafted	347.0 PdI = 0.37	58 (1%), 347 (96%), 5386 (3%)	56 (29%), 159 (40%), 470 (19%)	−31.1

## Data Availability

The original contributions presented in this study are included in the article. Further inquiries can be directed to the corresponding author.

## Abbreviations

The following abbreviations are used in this manuscript:

PI	3-hydroxy-2-methoxy-1,2-diphenylpropan-1-one
P(MMA-co-MA)	poly(methyl methacrylate-co-maleic anhydride)
MPI	poly(methyl methacrylate-co-maleic anhydride) with 3-hydroxy-2-methoxy-1,2-diphenylpropan-1-one
